# Giant Asymptomatic Submandibular Sialolith: A Case Report Accompanied by Systematic Review

**DOI:** 10.3390/clinpract15110205

**Published:** 2025-11-10

**Authors:** Renato Gomes Azevedo, Luan Felipe Toro, Vinícius Franzão Ganzaroli, Vinícius José Ifanger, Nathan Ayres de Faria, Rodrigo Ubiali de Rezende, Julia da Conceição Francisquini, Gestter Willian Lattari Tessarin

**Affiliations:** 1 School of Dentistry, University Center in the North of São Paulo (UNORTE), São José Do Rio Preto 15020-040, São Paulo, Brazil; renatogazevedo@hotmail.com (R.G.A.); viniciusifanger12@hotmail.com (V.J.I.); nathanfaria16@hotmail.com (N.A.d.F.); rodrigoubiali@outlook.com (R.U.d.R.); drajuliafrancisquini@gmail.com (J.d.C.F.); 2 Department of Basic Subjects, Marilia Medical School (FAMEMA), Marília 17519-030, São Paulo, Brazil; luan.toro@unesp.br; 3 Department of Basic Sciences, School of Dentistry, Sao Paulo State University (UNESP), Araçatuba 16015-050, São Paulo, Brazil; vinicius.ganzaroli@unesp.br

**Keywords:** asymptomatic, case report, giant sialolith, systematic review, Wharton’s duct

## Abstract

Background/Objectives: Salivary stones, also known as sialoliths, are calcified structures that develop within the salivary glands and/or their ducts. They occur in approximately 1 per 10,000 to 30,000 individuals per year, primarily affecting adults between 30 and 50 years of age. Although several hypotheses have been proposed, the exact mechanisms of formation and their predisposing factors are yet to be confirmed. The submandibular gland is the most commonly affected site, accounting for nearly 80% of cases, while giant and asymptomatic sialoliths are rare clinical findings in dental practice. This study is divided into two components: first, a case report of a giant, asymptomatic sialolith located in Wharton’s duct; second, a systematic review of the literature to explore the clinical procedures, diagnoses, outcomes, and other relevant aspects of this pathology. Methods: The case involved a 42-year-old woman who sought dental care due to the presence of a painless sublingual swelling. Intraoral examination and imaging revealed a calcified mass consistent with sialolithiasis in Wharton’s duct. The stone was successfully removed via sialolithotomy. For the systematic review, an extensive search was conducted in PubMed, Embase, and Cochrane Library up to June 2025, using specific keywords. Initially, 262 studies were identified. After applying inclusion and exclusion criteria, six case reports were included in the final analysis. Results: All selected studies described giant salivary stones located in Wharton’s duct and/or the parenchyma of the submandibular gland, notably without associated pain. Computed tomography and ultrasonography were the most commonly used imaging modalities for diagnosis. In all cases, the primary treatment was sialolithotomy. Conclusions: This study explored a rare case report of an asymptomatic giant sialolith in Wharton’s duct, and it includes a systematic review focused exclusively on asymptomatic giant sialoliths. It specifically addresses key characteristics, preferred imaging modalities, treatment strategies, and clinical considerations for managing this uncommon condition. Registration number: Prospero registration nº CRD420251076737.

## 1. Introduction

Asymptomatic giant sialoliths of the submandibular gland or Wharton’s duct, though rare, pose diagnostic and management challenges [1,2]. They represent the most common salivary gland disorder, accounting for approximately 30% of cases, with an incidence of 1 per 10,000–30,000 individuals per year. Most cases occur between the third and sixth decades of life, with a higher prevalence in males [1,2]. This pathology is connected to the accumulation of calcified material into the salivary ducts and/or glands [2,3], with the submandibular gland being the most frequently affected site (about 80–90% of all cases). This predominance is attributed to both anatomical and physiological factors, including the viscous and alkaline nature of its saliva, the long and upward course of Wharton’s duct, and the slower salivary flow compared to other salivary glands [2,4].

Typically, sialoliths present with painful swelling in the region of the affected gland and may lead to obstructed salivary outflow, infection, dysphagia, difficulty speaking, and, in some cases, ulceration of the oral mucosa [5,6]. This is particularly impactful during meals, as the obstructed salivary flow at that time, often referred to as “mealtime syndrome” [2,7], significantly worsens the patient’s quality of life [2]. Nonetheless, sialoliths can be asymptomatic, especially when the obstruction of the salivary gland duct is partial [8,9,10].

The pathogenesis of sialoliths is believed to involve the formation of a central organic nidus—such as desquamated epithelial cells or bacteria—around which inorganic salts, primarily calcium phosphate and hydroxyapatite, are deposited in concentric layers [3,11]. Furthermore, most sialoliths measure between 5 and 10 mm (mm); those exceeding 15 mm are classified as “giant sialoliths” [12,13,14]. Gadve et al. (2016) [4] emphasize that these account for only 7.6% of cases, highlighting how unusual these larger stones are [4].

Treatment depends on the specific salivary gland involved, as well as the size and anatomical location of the sialolith. For small calculi, conservative management is typically recommended and may include measures such as adequate systemic hydration, application of moist heat, glandular massage, sialogogue stimulation, and ductal dilation with catheterization to facilitate the removal of stones located near the ductal orifice. In contrast, larger sialoliths, or those situated within the glandular parenchyma, often require surgical intervention via intraoral access [15,16,17].

In the present study, we first describe the case of a female patient with an unusually large, asymptomatic sialolith located in Wharton’s duct. Additionally, we conducted a systematic review exploring asymptomatic sialoliths in the submandibular gland. Currently, there is a limited number of studies addressing the characteristics, prevalence, location, diagnostic methods, and other aspects of sialoliths. Thus, a broader and more rigorous review of the literature is needed to provide an updated and comprehensive understanding of this condition.

## 2. Case Report Presentation

This case report was prepared in accordance with the CARE (CAse REport) guidelines to ensure that the information provided is transparent, comprehensive, and reliable. All essential components recommended by the checklist have been included, such as all-encompassing patient information (age, sex, medical history, and relevant background), clinical presentation and diagnostic workup, including imaging findings, and a detailed description of the therapeutic intervention performed. The outcome and follow-up data were clearly described, and relevant aspects of consent were also addressed. By adhering to these standards, we aimed to enhance the clarity, reproducibility, and educational value of this case report.

A 42-year-old female patient sought comprehensive care at the Division of Dentistry, Northern University Center of São Paulo, presenting with a painless swelling in the right sublingual region that had persisted for over 30 days. The patient reported no episodes of pain, even during or after meals. Her medical history was unremarkable, with no use of medications, tobacco, alcohol, or other irritants, and no prior history of sialadenitis (Supplementary Material S1). Notably, the patient reported having experienced xerostomia (dry mouth) for approximately 20 days.

**Figure 1:**
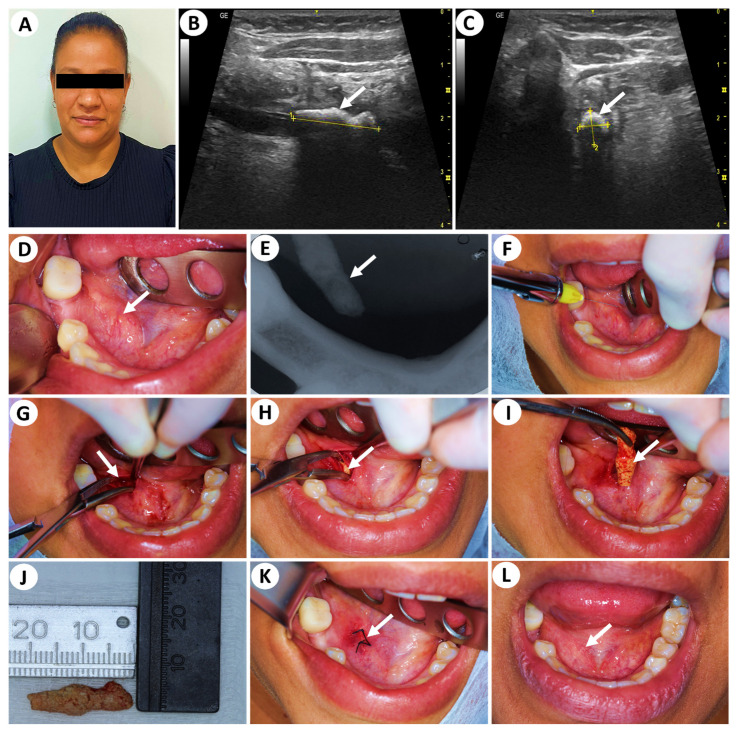
Patient profile (**A**), ultrasound exams (**B**,**C**), initial intraoral aspect (**D**), initial radiographic exam (**E**), sequential approach for removal of giant sialolith (**F**–**K**) and final aspect after surgical treatment (**L**). In A, absence of abnormalities extraoral. In (**B**,**C**) (white arrows), oval and hyperechoic image located in the distal topography of the right of Wharton’s duct. In (**D**), ovoid and well-defined submucosal swelling with normal color and texture (white arrow). In (**E**), radiographic aspect showing a mass along the internal surface of the right mandible (white arrow). In (**F**), local anesthesia (using articaine hydrochloride with epinephrine; DFL Indústria e Comércio, Rio de Janeiro, RJ, Brazil) technique. In (**G**), incision and divulsion (white arrow). In (**H**), divulsion and dissection of tissues to locate the giant sialolith (white arrow). In (**I**), removal of the giant sialolith (white arrow shows the giant sialolith). In (**J**), the length (21 mm) and the width (9 mm) of the giant sialolith. In (**K**), sutured tissues using 4-0 silk (white arrow; Ethicon, Johnson and Johnson do Brasil, São José dos Campos, SP, Brazil). In (**L**), absence of swelling and normal appearance of the oral mucosa seven days postoperatively (white arrow).

Clinical examination revealed no abnormalities in the structures of the face, head, and/or neck (Figure 1A). Intraoral inspection showed a firm, ovoid, and well-defined submucosal swelling, measuring approximately 25 × 15 mm (length × width), located in the floor of the mouth, adjacent to the right sublingual caruncle (Figure 1D). The overlying mucosa was intact, with normal color and texture, and no signs of inflammation or infection were observed (Figure 1D).

For diagnostic continuity, a periapical radiographic film (30.5 × 40.5 mm) was positioned in the region of the lower right premolar and molar teeth to obtain an occlusal radiograph aspect. The imaging revealed a sharply delineated radiopaque mass along the internal surface of the right mandible (Figure 1E). Based on the initial evaluations, including anamnesis, clinical examination, and preliminary imaging, the findings were consistent with a giant sialolith located in the right submandibular triangle. Following the initial assessment, the patient was referred to an ultrasonographic evaluation to obtain a more precise diagnosis, including measurement, anatomical localization, and assessment of the mass’s proximity to vital structures. The ultrasound examination revealed the presence of oval and hyperechoic image, determining posterior acoustic shadows located in the distal topography of the right of Wharton’s duct, measuring approximately 20 × 6 × 6 mm (length × width × thickness) (Figure 1B,C; Supplementary Material S2). Additionally, blood and serum biochemical analyses were within normal limits.

Following informed consent (Supplementary Material S1), the patient underwent a surgical procedure for the removal of the giant sialolith. Oral antisepsis was performed using 0.12% chlorhexidine gluconate (Colgate-Palmolive, São Bernardo do Campo, São Paulo, Brazil) for one minute. Local anesthesia was administered using articaine hydrochloride (72 mg/carpule) with epinephrine (18 µg/carpule) (Figure 1F; DFL Indústria e Comércio, Rio de Janeiro, RJ, Brazil). A linear incision, about 20 mm long, was made in the right floor of the mouth using a 15C blade attached to a scalpel handle, and the soft tissues were carefully dissected to expose Wharton’s duct (Figure 1G). The giant sialolith was then gently accessed and removed in one piece from the surgical site (Figure 1H,I). Macroscopic examination revealed a hard, yellowish stone measuring 21 mm in length and 9 mm in width (Figure 1J). After removal, the surgical site was sutured using 4-0 silk sutures (Ethicon, Johnson and Johnson do Brasil, São José dos Campos, SP, Brazil) (Figure 1K). The following postoperative medications were prescribed: antibiotics (amoxicillin 1500 mg/day fractioned in 3 times during 7 days), analgesics (dipyrone sodium, 1500 mg/day fractioned in 3 times during 3 days), and anti-inflammatories (nimesulide 200mg/day fractioned in 2 times during 3 days). It is important to note that the postoperative medication regimen reported here reflects our local clinical practice and may vary according to institutional protocols and regional guidelines.

Finally, Institutional Review Board (IRB) approval was waived due to the descriptive nature of a single case report; written informed consent for publication was obtained.

### Follow-Up

The patient was reevaluated on the seventh postoperative day, and a clinical examination revealed a normal right floor of the mouth and adequate salivary flow through the healed ductal orifice (Figure 1L). In addition, the patient still reported no pain, as was the case when they first visited the specialized university team. It is noteworthy that the patient also reported that the feeling of “dry mouth” had disappeared, and food had been tasting better. To ensure the complete resolution of this case, the patient was reevaluated thirty days after surgery, and once again, their aspect was normal.

## 3. Materials and Methods Applied in the Systematic Review

### 3.1. Protocol and Registration

This systematic review was registered in the International Prospective Register of Systematic Reviews (PROSPERO), under the registration number CRD420251076737 (Supplementary Material S3). The research question was structured using the PEO framework (Population, Exposure, Outcome) as follows:

- P (Population): Individuals with giant sialoliths (defined as stones larger than 15 mm);
- E (Exposure): Presence of giant and asymptomatic sialoliths in the submandibular gland or Wharton’s duct;
- O (Outcome): Clinical presentation, diagnostic approaches, treatment modalities, and postoperative outcomes from case reports.

Accordingly, the primary research question addressed in this review was:

“In patients with submandibular sialolithiasis (P), how does the presence of a giant asymptomatic sialolith (E) influence the clinical presentation, diagnostic approach, management, and treatment outcomes reported in literature?”

This systematic review was conducted and reported in accordance with the Preferred Reporting Items for Systematic Reviews and Meta-Analyses (PRISMA) 2020 statement [18], and the completed PRISMA checklist is provided as Supplementary Material S4.

### 3.2. Literature Search Strategy

*Step 1: Specialized Search*

An exhaustive and structured literature search was conducted by independent and blinded reviewers (G.W.L.T. and R.G.A.) across the PubMed, Embase, and Cochrane Library electronic databases. Based on the selected descriptors, three other researchers (V.J.I.; N.A.F.; R.U.R.) independently searched the case reports to ensure data security, after which the data was compared. The search strategy employed Boolean operators (AND and/or OR) in combination with DeCS/MeSH-controlled vocabulary terms, including: “asymptomatic”, “giant calculi”, “giant sialolith”, “giant sialolithiasis”, “giant submandibular stone”, “submandibular duct”, “submandibular gland”, “submandibular salivary gland”, and “Wharton’s duct”. Specifically, in the electronic literature libraries cited above, the complete reproducible search combinations used were: asymptomatic and(or) giant calculi and(or) submandibular duct; asymptomatic and(or) giant calculi and(or) submandibular gland; asymptomatic and(or) giant calculi and(or) submandibular salivary gland; asymptomatic and(or) giant calculi and(or) Wharton’s duct; asymptomatic and(or) giant sialolith and(or) submandibular duct; asymptomatic and(or) giant sialolith and(or) submandibular gland; asymptomatic and(or) giant sialolith and(or) submandibular salivary gland; asymptomatic and(or) giant sialolith and(or) Wharton’s duct; asymptomatic and(or) giant sialolithiasis and(or) submandibular duct; asymptomatic and(or) giant sialolithiasis and(or) submandibular gland; asymptomatic and(or) giant sialolithiasis and(or) submandibular salivary gland; asymptomatic and(or) giant sialolithiasis and(or) Wharton’s duct; asymptomatic and(or) giant submandibular stone and(or) submandibular duct; asymptomatic and(or) giant submandibular stone and(or) submandibular gland; asymptomatic and(or) giant submandibular stone and(or) submandibular salivary gland; asymptomatic and(or) giant submandibular stone and(or) Wharton’s duct.

In all electronic literature databases, the search for studies included and excluded in this review considered publications up to August 2025. It is important to emphasize that these studies were identified after the protocol was registered on the PROSPERO platform, thereby characterizing this work as a prospective systematic review.

*Step 2: Identification of studies*

Studies were selected based on previously established eligibility criteria considering the scope of this study. Inclusion criteria: (1) only articles published in English were considered eligible for inclusion. This restriction was applied because English is the predominant language of publication in biomedical sciences, especially for case reports in oral and maxillofacial pathology, and because it allowed our team to ensure consistency and accuracy in data extraction and interpretation; (2) there was no restriction regarding the date of publication of the studies; (3) only case reports were considered; (4) only cases involving asymptomatic giant sialoliths (more than 15 mm) located in the submandibular gland and/or Wharton’s duct. Exclusion criteria: (1) manuscripts not published in English; (2) conference abstracts; (3) case reports describing painful or symptomatic presentations; (4) studies that are not case reports; (5) reports of sialoliths smaller than 15 mm. Disagreements between reviewers were resolved through consensus.

*Step 3: Screening of studies*

During this phase, duplicate records were identified and removed. The remaining articles were then screened based on their titles and abstracts, applying the predefined inclusion and exclusion criteria. All steps were conducted manually by two independent reviewers (G.W.L.T. and R.G.A.). Disagreements were resolved through discussion with a third reviewer (L.F.T). The Rayyan platform (https://www.rayyan.ai) (Accessed on 30 July 2025) was used to double-check the screening selection process.

*Step 4: Inclusion and analysis of studies*

In the final stage, studies that met all eligibility criteria were retrieved for full-text reviews. Relevant data [such as sex/age (years), side involved, images for diagnosis, location of sialolith, size (mm), swelling, and management] were extracted from these articles, and a systematic review was subsequently developed, synthesizing the most pertinent evidence related to asymptomatic giant sialoliths.

### 3.3. Standardization andQuality of Studie, Risk of Bias and Analysis

To ensure transparency and consistency in this study, the CARE (CAse REport) checklist was descriptively applied to verify the completeness of information presented in the included case reports. The checklist was not used as a scoring or quality assessment tool. It comprises 30 key items designed to promote transparent and comprehensive documentation of case reports. Each item was evaluated as “YES” (Y; adequately reported) or “NO” (N; partially reported or not reported). Two independent reviewers (G.W.L.T. and R.G.A.) assessed each report, and in cases of disagreement, a third reviewer (L.F.T.) made the final decision. The final analysis presented the absolute numbers for YES (Yn) or NO (Nn) and percentages of Y (%Y) and N (%N) responses.

In addition, two blinded and independent reviewers (G.W.L.T. and R.G.A.) assessed the methodological quality of the included studies using the Joanna Briggs Institute (JBI) Critical Appraisal Checklists for Case Reports and Case Series (https://joannabriggs.org/). Each of the eight items on the JBI tool was scored as ‘Yes’, ‘No’, ‘Unclear’, or ‘Not applicable’. A score of one (1) point was assigned for each ‘Yes’ response, while ‘No’ and ‘Unclear’ responses received zero (0). The maximum possible score was eight, and final scores were converted into percentages, ranging from 0% to 100%. Based on these percentages, studies were categorized as having high (80–100%), moderate (50–79%), or low (<50%) methodological quality. In cases of disagreement, a third reviewer (L.F.T.) made the final decision.

## 4. Results

**Figure 2:**
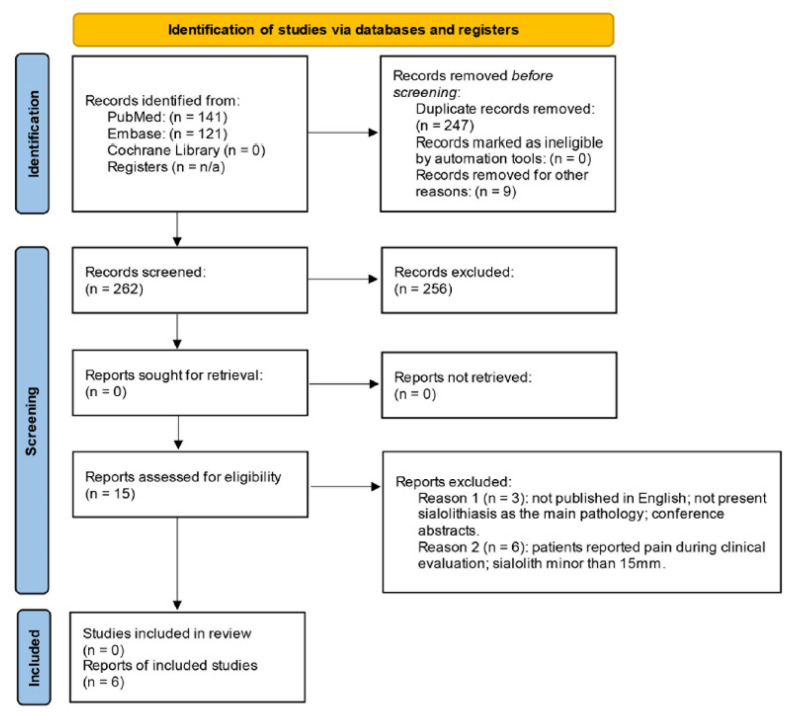
PRISMA flow diagram of study search and identification of relevant studies. Abbreviation: n/a, not applicable.

As part of the literature search strategy, a total of 262 articles were identified: 141 from PubMed, 121 from Embase, and 0 from the Cochrane Library. 247 duplicates were excluded, and the remaining 15 were initially selected for a full screening. 3 of them were excluded because they were not published in English, did not present sialolithiasis as the main pathology, or were conference abstracts. Subsequently, 6 studies were excluded, as they were not in consonance with eligibility criteria or because the patients reported pain during clinical evaluation. Ultimately, 6 studies met all eligibility criteria and were included in this systematic review (Figure 2). Details regarding authorship, affected side, patient age and sex, image used for diagnosis, and management strategies are summarized in Table 1.

### 4.1. Demographics Data

The frequency of asymptomatic giant sialoliths was found to be higher in men than in women. Only one of the six studies included in this systematic review reported the occurrence of this asymptomatic condition in a female patient, while the remaining five described male patients. The mean age of the patients was 57.2 years (±13.1; median: 60 years), with an age range from 37 to 75 years. The studies included were conducted across three continents: Asia (Turkey, Taiwan, Saudi Arabia), America (United States), and Europe (Italy and Turkey). It is important to note that Turkey was cited as part of both Asia and Europe, due to its unique geographical position, lying partly in Asia and partly in Europe. Among the selected studies, only Graziani et al. (2006) [19] explicitly reported the ethnicity of the patient as Caucasian; the remaining studies did not provide information regarding ethnic background.

### 4.2. Characteristics of the Included Case Reports and Follow-Up

As required by the inclusion criteria, no patient in the selected studies reported pain during anamnesis. Only one study (16.7%)—Graziani et al. (2006) [19]—reported no swelling in the floor of the mouth, while the remaining five studies (83.3%) [1,8,20,21,22] described the presence of swelling in the submandibular region or floor of the mouth. Both the left and right sides of the mouth were affected across the cases. Emir et al. (2010) [20] described a giant, fistulized sialolith causing swelling on the right side of the floor of the mouth and noted the presence of multiple smaller calculi on the left side, which did not cause swelling. Cottrell et al. (2011) [8], Abdullah and Alqudehy (2016) [21], and Liu and Lo (2023) [22] all reported sialoliths located on the left side. Brooks et al. (2021) [1] described swelling near the left sublingual caruncle, along with a diffusely bordered radiopaque mass in the anterior mandibular region, though without associated swelling. In summary, 66,7% (4 studies; [1,8,21,22]) of the cases reported swelling on the left side; in 16,7% (1 study; [20]) of cases, the swelling was observed on the right side; 16,7% (1 study; [19]) of cases did not report any swelling.

Regarding imaging exams used for diagnosis, computed tomography (CT) was among the most employed modalities, used in four studies [1,19,21,22], followed by panoramic radiographies, reported in three studies [1,8,19]. Additional diagnostic tools included ultrasound [19,21], periapical radiographies [1], and occlusal radiographies [8]. It is important to highlight that Graziani et al. (2006) [19], Cottrell et al. (2011) [8], Abdullah and Alqudehy (2016) [21], and Brooks et al. (2021) [1] employed more than one imaging modality to establish the diagnosis of giant sialolithiasis, enhancing diagnostic accuracy. Emir et al. (2010) [20] noticed the use of plain radiography and ultrasonography as complementary exams for the diagnosis of salivary stones.

About the location of the giant sialoliths, 50.0% of the studies [19,21,22] reported that the salivary stones were located in Wharton’s duct, while 33.3% [1,20] described the presence of sialoliths within the parenchyma/stroma of the submandibular gland. Cottrell et al. (2011) [8] 16.7% did not provide precise information regarding the anatomical location of the salivary stone.

After diagnosis and treatment planning, 83.3% of the selected studies [8,16,20,21,22] opted for surgical removal of the salivary stone. Additionally, Abdullah and Alqudehy (2016) [21] performed marsupialization of Wharton’s duct. Brooks et al. (2021) [1] reported that the patient did not present with xerostomia, episodic swelling, or pain; the patient declined any further diagnostic procedures and was subsequently lost to follow-up. Liu and Lo (2023) [22] and Abdullah and Alqudehy (2016) [21] performed the surgical procedure under general anesthesia, whereas Cottrell et al. (2011) [8], Graziani et al. (2006) [19], and Emir et al. (2010) [20] opted for local anesthesia. The anesthetic approach was not described by Brooks et al. (2021) [1], as the patient refused further evaluation and treatment. None of the studies specifically mentioned prescribing medications before or after the surgical intervention for sialolithiasis. However, Abdullah and Alqudehy (2016) [21] reported the use of intravenous antibiotics (unspecified) for 10 days prior to intraoral removal of the left submandibular stone, as the sialolithiasis was associated with abscess formation.

All sialoliths removed through surgical approaches measured more than 15 mm in length, thus qualifying as giant sialoliths. All included studies reported the length of the salivary stones, with a mean extension of 30.56 mm (±5.5 mm; median: 32 mm; range: 22–36 mm). Five studies also described the width of the stones, with a mean of 19.10 mm (±10.0 mm; median: 18.5 mm; range: 8–35 mm). Only one study (Cottrell et al., 2011 [8]) reported the thickness, which was 20.00 mm. It is important to note that Emir et al. (2010) [20], Cottrell et al. (2011) [8], and Brooks et al. (2021) [1] also reported the presence of additional sialoliths beyond the main giant calculus described. Finally, five studies reported that the surgical removal of the stones resulted in the resolution of the pathology associated with the salivary gland and/or ductal system. As described above, Brooks et al. (2021) [1] did not describe the follow-up, as the patient was lost and refused any type of treatment.

Table 1 summarizes the main findings described.

**Table 1:** Summary of all studies included in this systematic review.

Author	Country	Sex/Age (years)	Side Involved	Images for Diagnosis	Location of Sialolith	Size (mm)	Swelling	Pain	Anesthesia	Management	Follow-up
Liu and Lo, 2023 [22]	Taiwan	Female/48	Side Involved	Tomography	Wharton’s duct	34 × 14	Yes	No	General	Surgical Removal	No complications and good recovery
Brooks et al., 2021 [1]	USA	Male/63	Left	Panoramic Radiograph, Periapical Radiograph and Tomography	Submandibular Gland and Duct	26.4 × 18.5	Yes	No	No reported ^#^	Preservation *	Preservation *
Abdullah and Alqudehy, 2016 [21]	Saudi Arabia	Male/37	Left	Ultrasound and Tomography	Wharton’s duct	36	Yes	No	General	Surgical removal and marsupialization of the duct	Smooth recovery with no complications
Cottrell et al., 2011 [8]	USA	Male/75	Left	Panoramic Radiograph and Occlusal Radiograph	Wharton’s duct	30 × 20 × 20	Yes	No	Local	Surgical Removal	No Swelling, redness, erythema, or signs of infection,
Emir et al., 2010 [20]	Turkey	Male/59	Left	Panoramic Radiograph and Ultrasound	Submandibular Gland	35 × 35	Yes	No	Local	Surgical Removal	Successful healing without complications
Graziani et al., 2006 [19]	Italy	Male/61	Right and Left	Panoramic Radiograph,	Wharton’s duct	22 × 8	No	No	Local	Surgical Removal	No complications and normal salivary flux.

* Brooks et al. 2021 [1], described that, since their patient had not elicited any apparent xerostomia, episodic swelling or pain, he declined any additional diagnostic measures and was lost to follow-up. # Brooks et al. (2021) [1] reported that the patient did not present with swelling, xerostomia, or pain. Therefore, no intervention was undertaken to resolve the case, and consequently, no anesthetic technique was described or employed.

### 4.3. Standardization and Quality of Studies and Risk of Bias

All case reports (*n* = 6; [1,8,19,20,21,22]) included in this systematic review presented reasonable compliance and risk of bias (Table 2 and Table 3). The studies were published in scientific journals (the *Journal of craniofacial surgery*, Graziani et al., 2006 [19], *Ear, nose, & throat journal*; Emir et al., 2010 [20], *Journal of the Massachusetts Dental Society*; Cottrell et al., 2011 [8], *Indian Journal of Otology*; Abdullah and AlQudehy, 2016 [21], *Gerodontology*; Brook et al., 2021 [1], *Ear, nose, & throat journal*; Liu and Lo et al., 2023 [22], *Ear, nose, & throat journal*) and were accepted following peer review. A descriptive analysis of the included case reports was performed using the CARE checklist criteria. Overall, compliance with CARE items varied across the studies. Four studies [8,19,21,22] demonstrated a compliance index greater than 50.0%. In contrast, Brooks et al. (2021) [1] showed a compliance index of 43.3%, and Emir et al. (2010) [20] reported 46.7% (Table 2). Nevertheless, all studies were classified as having a moderate risk of bias, according to the Joanna Briggs Institute (JBI) Critical Appraisal Checklists for Case Reports and Case Series (Table 3).

## 5. Discussion

Giant sialoliths in the submandibular gland are rare entities, and their asymptomatic presentation is even more exceptional. Typically, sialolithiasis causes intermittent pain and swelling due to obstructed salivary flow, particularly during meals. However, in the present case report, the patient exhibited no pain, despite the size and duration of the swelling, suggesting that partial ductal patency and adaptive glandular responses may have prevented symptomatic obstruction. In addition, to our knowledge, no prior systematic review has focused exclusively on asymptomatic giant sialoliths or aimed to provide a comprehensive synthesis of the existing literature on asymptomatic giant sialoliths or offer an up-to-date overview of their main features, including clinical implications, management strategies, and outcomes. In summary, a total of six studies (all case reports: [1,8,19,20,21,22]) were included. All selected studies reported the presence of asymptomatic giant sialoliths located either in the submandibular gland or in Wharton’s duct.

### 5.1. Pathogenesis and Hypotheses for Asymptomatic Presentation

Sialolithiasis is the most common disease affecting the salivary glands, with a higher prevalence in middle-aged men [23]. Typically, sialoliths cause pain and/or swelling in the oral cavity, symptoms that are often exacerbated during meals due to increased salivary flow [24,25]. Clinical manifestations can range from mild discomfort to severe pain and may be accompanied by cervical swelling or trismus. However, some cases remain completely asymptomatic [22], challenging the conventional diagnostic paradigm and raising important questions regarding pathophysiology, detection, and clinical management [26]. Several hypotheses have been proposed to explain the absence of symptoms in certain patients [3,12,17,22,27,28,29]. First, the lack of pain may be related to the size of the stone [3,9,17]. Smaller sialoliths may cause only partial obstruction of the salivary duct, allowing saliva to flow around the stone and preventing pressure buildup and the subsequent development of pain or swelling [3,9,12,17]. Second, chronic obstruction by sialoliths—even without overt infection—may lead to progressive glandular atrophy, loss of secretory function, and fibrosis, ultimately resulting in asymptomatic chronic sialadenitis over time [22,28,30]. Third, estimates suggest that sialoliths grow at a rate of 1–1.5 mm per year. This slow progression may allow for gradual ductal dilation, thereby maintaining near-normal salivary flow and contributing to an asymptomatic clinical course [27,29]. In the case presented here, the patient reported a complete absence of pain, and all studies included in this systematic review likewise described painless presentations. The aforementioned hypotheses are potential explanations for the absence of pain in our patient and the other cases reviewed.

A sialolith or salivary stone is a calcified structure that forms within the ductal system or parenchyma of a salivary gland. Sialoliths are primarily composed of inorganic minerals such as calcium phosphate (hydroxyapatite) and organic substances, including glycoproteins, cellular debris, and mucins. Their formation is believed to result from a combination of salivary stasis, altered composition of saliva, ductal injury, or infection, which leads to precipitation of salts around a central nidus [31,32,33]. Among the major (parotid, submandibular, and sublingual glands) salivary glands, the submandibular gland is the most frequently involved [1,34]. Anatomical distribution studies have shown that approximately 53% of submandibular gland sialoliths are located in the hilar or proximal region of Wharton’s duct; 37% in the distal portion of the duct, and 10% within the glandular parenchyma [1,7]. This can be due to a combination of anatomical, physiological, and biochemical factors that predispose it to salivary stasis and crystal precipitation [12,32,35]. Anatomical characteristics, such as length (average of 5–6 cm) and curvature, ensure that the saliva follows an upward path from the deep portion of the gland to its orifice at the sublingual caruncle, and this antigravity trajectory makes salivary drainage more difficult, increasing the risk of stagnation and precipitation of minerals [12,35]. In addition, it can contribute to greater mineral saturation and increased risk of sialolithiasis, including the high concentration of calcium and phosphate ions in submandibular saliva compared to other glands, and the potential migration of food particles, bacteria, or foreign bodies into the ductal system from the oral cavity, serving as nucleating agents for calcification [3,32,36]. The elevated salivary alkalinity of the submandibular gland can be another factor that contributes to stone formation [1]. In the case reported here, the salivary stone was located in Wharton’s duct, as was the case for most of the studies selected for this systematic review. The factors discussed may contribute to the formation of stones in this anatomical region.

### 5.2. Diagnostic Imaging

For the diagnosis and location of giant sialoliths, a variety of imaging exams can be used [1,8,17,19,21,37,38]. Occlusal radiographies used to be the initial diagnostic of choice, but this exam is only effective in detecting large radiopaque sialoliths within the ductal system, being limited in its capability of identifying smaller or intraparenchymal stones. In addition, as approximately 20% of sialoliths are radiolucent, a significant number may go undiagnosed using standard radiography alone. Although conventional imaging remains useful in the initial evaluation, modern modalities such as ultrasonography and computed tomography (CT) offer superior diagnostic accuracy, allowing for precise localization and characterization of salivary stones [17,39]. Non-contrast computed tomography (NCCT) is an effective tool for the diagnosis of sialoliths when they reach a sufficient size or when high-resolution imaging with thin-slice (e.g., 1 mm) intervals is employed. In addition, the NCCT offers high sensitivity and specificity for detecting calcified sialoliths, along with rapid image acquisition and broad availability. Nonetheless, its limitations include exposure to ionizing radiation, in addition to suboptimal assessment of the ductal system and associated soft tissue pathologies [17,40,41,42]. Ultrasonography, in turn, is a non-invasive method of imaging for diagnosis of sialolithiasis and is regarded as the go-to imaging exam in many clinical settings worldwide, due to its widespread availability, cost-effectiveness, and no need for ionizing [19,21,38]. Studies have reported that this technique has better applicability in terms of sensitivity and specificity for stones larger than 2 to 3 mm in size [17,43]. In our case report, we used radiography and ultrasound as image exams for the diagnosis of a giant sialolith, and the selected studies included in our systematic review also used radiography in addition to tomography and/or ultrasound. A study conducted by Thomas et al. (2017) [44], which analyzed the accuracy of ultrasonography and computed tomography in patients with sialolithiasis, observed that the sensitivity of ultrasound was 65% and the specificity for diagnosis was 80% [44]. Similarly, Özçelik et al. (2024) [38] determined the sensitivity of ultrasound to be 88% [38], finding that computer tomography is more sensitive (95%; 59 detected of 62 stones) than ultrasound (74%; 46 detected of 62 stones) in detecting distal ductal stones in the submandibular gland [38]. Likewise, Thomas et al. (2017) [44] determined that computed tomography was more sensitive and specific (98% and 88%, respectively) for the diagnosis of sialoliths in the major salivary glands [44]. Thus, we can infer that radiographies could be used for the first diagnosis of sialoliths, but ultrasound and computer tomography were complementary to diagnose with precision the location, size, and other characteristics of sialoliths in salivary glands to optimize the outcome and promote a specific treatment selection for the resolution of this type of pathology.

### 5.3. Considerations and Directions to Treatment

The treatment of salivary gland sialoliths depends on the number, location, size, composition of the stone, symptoms, and the affected gland [17,45]. Initial management is typically conservative, including glandular massage, anti-inflammatory drugs, and sialogogues to stimulate salivary flow [16,46,47,48]. If conservative measures fail, alternative interventions are indicated [17]. Specifically, for submandibular sialoliths in Wharton’s duct, mobile stones <5 mm in the distal duct are best managed initially with sialendoscopy [17,45,49,50,51]. Impacted distal stones or those >5 mm usually require transoral ductal slitting, while stones 5–7 mm in the proximal duct or hilar region are first approached endoscopically; transoral surgical removal is indicated if endoscopy fails [17,45,49,50,51]. Additionally, laser lithotripsy, extracorporeal shockwave lithotripsy (ESWL), and transoral robotic surgery (TORS) may be considered for giant sialoliths [17,45,52,53,54]. These techniques will be further discussed in Section 5.5.

Before deciding on a treatment for salivary stone removal, however, the locoregional anatomy [16,21,55] must be considered. According to Marchal et al. (2001) [30] and Liu and Lo (2023) [22], transoral sialolithotomy with sialodochoplasty or sialadenectomy is still the mainstay for the management of giant sialoliths [22,30]. In the present case report, transoral sialolithotomy was selected as the therapeutic approach, as it is a minimally invasive technique for the removal of giant sialoliths, which helps minimize the risk of associated complications. Furthermore, all studies selected for the systematic literature review (except study [1]) presented stones located in Wharton’s duct [8,19,22] or within the submandibular gland [20], and surgical removal of the sialolith was the treatment of choice.

### 5.4. Strengths, Methodological Considerations and Limitations of This Study

#### 5.4.1. Strengths and Methodological Considerations

This study presents several noteworthy strengths. To our knowledge, this study is a pioneer in associating a rare case of a giant sialolith within Wharton’s duct with a systematic review focused specifically on this pathology. Moreover, all case reports included in the review were assessed using both the CARE and Joanna Briggs Institute (JBI) Critical Appraisal Checklists to ensure consistency, standardization, compliance, and methodological rigor. This comprehensive evaluation allows readers to critically appraise the quality of the studies included in this review. The study design also incorporated an exhaustive literature search, clearly defined eligibility criteria, and rigorous procedures for study screening and data extraction, thereby enhancing transparency and reproducibility. Finally, the use of two (or three, in the event of a tie) independent reviewers for preselection, quality assessment, and data extraction minimized errors and reduced potential subjective bias.

Case reports document a patient’s medical condition and its clinical management to contribute to scientific knowledge or medical education. Historically, they have been crucial for identifying rare or novel diseases, evaluating the benefits and risks of interventions, and enhancing medical education through detailed real-world examples [56]. The CARE Checklist, which outlines the essential information that needs to be included when writing a case report, provides a structured framework that can be adapted to incorporate specialty-specific details, and is designed to enhance the overall quality, compliance and reporting standards of case reports [56]. The Joanna Briggs Institute (JBI) Critical Appraisal Checklist for Systematic Reviews and Research Syntheses offers a concise and effective tool for evaluating the methodological rigor of reviews and identifying strategies employed by authors to minimize bias, and can be applied to analyze the transparency, reliability, and methodological quality of case reports [57]. Thus, two checklists (one for compliance and another for risk of bias) were applied to enable direct comparisons among the selected studies, assess the convergence of their findings, and strengthen the robustness of the systematic review.

Another strength of the present study lies in the documentation of a rare case of giant submandibular sialolith, providing detailed clinical, radiographic, and surgical information that can guide clinicians facing similar scenarios. Additionally, this is a pioneer systematic review focused exclusively on sialoliths larger than 15 mm, offering a comprehensive synthesis of the literature on their clinical presentation, diagnostic strategies, and management options.

#### 5.4.2. Limitations

This study has limitations. Our case report reflects a single clinical observation, which limits the generalizability of the findings. Additionally, the literature on giant sialoliths is scarce and mainly consists of isolated case reports and small case series, representing a low level of evidence. In addition, the literature search was conducted without date restrictions (completed in August 2025) across major databases (PubMed, Embase, and Cochrane Library), and only studies published in English were included. This language restriction may have excluded relevant studies in other languages, introducing potential linguistic bias.

The reliability of a systematic review fundamentally depends on the quality of the studies included [58]. Consequently, if the incorporated studies are of low quality, the conclusions drawn from the review may be less robust [58]. In our systematic review, only six studies were included. According to the CARE Checklist guidelines, four studies [8,19,21,22] demonstrated compliance indices greater than 50.0%, while the remaining two studies [1,20] showed indices above 43.0%. Additionally, all included studies exhibited a moderate risk of bias, as determined using the Joanna Briggs Institute Critical Appraisal Checklist for Case Reports. Although none of the studies achieved high compliance or low risk of bias, it is important to highlight that case reports offer several significant advantages [58]. They facilitate the identification of novel clinical observations, support the generation of new hypotheses, and contribute to pharmacovigilance [58]. Moreover, case reports are particularly valuable in contexts where more rigorous research designs are impractical or unethical [58]. Their narrative structure enables rich clinical detail and promotes educational value, fostering a deeper understanding of rare conditions. Therefore, despite its inherent limitations, this systematic review provides a meaningful contribution to the scientific understanding of giant sialoliths.

Another point that can be discussed is the implementation of a meta-analysis. It is important to note that meta-analyses, which combine data from multiple studies to improve precision and resolve conflicting results, require studies with adequate sample sizes. Applying meta-analytic methods to case reports is inappropriate, as they lack control groups, randomization, standardized follow-up, and comparable outcome measures. We conducted a systematic review with descriptive synthesis to summarize key clinical variables—such as demographics, stone size, sex distribution, treatments, location, and imaging—providing practical insights for dental practice [56,59,60,61].

### 5.5. Conflicting Findings and Minimally Invasive Alternatives

This systematic review explored the main clinical characteristics, diagnosis, treatment and other features linked to painless giant sialoliths. The number of studies that investigated this pathology is limited. However, some giant sialoliths located in Wharton’s duct and/or in the parenchyma/stroma of the submandibular gland cause pain. Chaidas et al. (2023) [62] described the presence of a giant salivary stone (58 mm × 17 mm) in Wharton’s duct, where the patient related pain and tenderness. Likewise, Abraham et al. (2021) [63], Sakthivel et al. (2017) [64] and Gadve et al. (2016) [4] reported pain on the floor of the mouth in patients diagnosed with giant sialoliths in the submandibular region. Thus, the giant salivary stones located in the submandibular gland and/or Wharton’s duct could be related to pain.

It is important to mention that salivary stones in submandibular salivary glands and/or their ductal system could be managed with minimally invasive techniques [31,44,50,65,66]. For instance, techniques such as sialendoscopy, laser lithotripsy, extracorporeal shockwave lithotripsy (ESWL), and transoral robotic surgery (TORS) [66,67,68,69] can be used. For example, sialendoscopy is a minimally invasive technique for the visualization and removal of submandibular sialoliths, offering the potential to reduce the risk of nerve injury, facial scarring, and oral trauma associated with conventional open surgical approaches [65,66,70,71]. Submandibular sialoliths measuring up to 5 mm in diameter can be effectively removed using sialendoscopy alone, particularly when the stones are mobile and freely located within the ductal lumen [67].

Another option is the use of sialendoscopy with laser lithotripsy. This technique consists of using a laser to break down stones that are too large for simple removal by sialendoscopy [67]. Submandibular sialoliths measuring between 5 and 7 mm can be fragmented within the ductal lumen using endoscopically guided laser lithotripsy prior to manual extraction [54]. Among the available techniques, holmium: YAG (yttrium–aluminum–garnet) laser-assisted lithotripsy is the most employed for salivary gland stones and has been demonstrated to be an effective, safe, and relatively straightforward method for managing larger submandibular sialoliths [72].

ESWL is a modality that employs focused high-energy shock waves generated externally to fragment salivary calculi into smaller particles [67]. These fragments can subsequently pass spontaneously through the ductal system or be removed with less invasive techniques, thereby reducing the need for conventional surgical intervention [67]. However, residual stone fragments often cannot be fully cleared by salivary flow, potentially leading to recurrence. For this reason, sialendoscopy is frequently performed following ESWL treatment. According to Capaccio et al. (2004) [73], complete clearance of residual stone fragments was achieved in 49% of cases with hilo-parenchymal stones and 28% of cases with stones located distally. Overall, the success rate of ESWL decreases with increasing stone size, and perihilar or intraparenchymal submandibular gland stones measuring less than 7 mm are considered the most suitable candidates for this technique [54,74].

Transoral robotic surgery (TORS) can be used to remove submandibular sialoliths, particularly those located in the hilar or parenchymal regions. This approach provides surgeons with enhanced three-dimensional visualization and precise instrument control via an intraoral route. In contrast to conventional open surgery, TORS eliminates the need for external cervical incisions, preserves anatomical structures (such as lingual nerve, hypoglossal nerve, and Wharton’s duct), and facilitates faster postoperative recovery, making it a valuable therapeutic option for patients with large or impacted salivary stones [69,75,76]. Lazzeroni et al. (2025) [69] carried out a systematic review and meta-analysis comparing TORS and conventional transoral surgery success to solve submandibular stone pathology, and they observed that transoral robotic surgery is a safe and effective modality for the management of submandibular gland sialoliths, demonstrating success and complication rates comparable to those of conventional approaches [69].

Specifically, for sialoliths larger than 15 mm, a combined approach—often referred to as sialendoscopy-assisted sialolithotomy—has emerged as the preferred minimally invasive strategy [77]. This technique integrates endoscopic guidance with a limited transoral incision, allowing for precise stone extraction while minimizing ductal trauma and preserving salivary gland function. In selected cases, particularly when the stone is located deeply within the hilum or parenchyma, transoral robotic surgery (TORS) has been proposed as an advanced and minimally invasive alternative, offering excellent visualization, reduced risk of lingual nerve injury, and high gland preservation rates [78]. Overall, these minimally invasive and gland-preserving strategies have shifted the therapeutic paradigm, reserving traditional sialadenectomy for rare cases in which other techniques are contraindicated or unsuccessful.

Finally, it is noteworthy that, in rare cases, giant sialoliths may be discharged spontaneously. Zhang et al. (2019) [79] reported a 29-year-old asymptomatic man in whom an intraoral examination revealed a cylindrical yellow mass at the orifice of the right submandibular duct. No imaging exams were performed. The sialolith was removed directly in the emergency room using mosquito forceps under local anesthesia, without any incision [79].

### 5.6. Brief Clinical Implications and Future Directions

Despite their often-dramatic size, giant submandibular sialoliths may remain clinically silent and undetected for prolonged periods. This highlights the importance of maintaining a high index of suspicion, especially when evaluating incidental radiographic findings in asymptomatic patients. Cross-sectional imaging modalities—particularly computed tomography (CT) and/or ultrasonography—are still the most effective tools for precise localization, size estimation, and surgical planning, outperforming conventional radiographies in both sensitivity and anatomical resolution. The absence of pain or infection should not delay intervention, as long-standing ductal obstruction can lead to progressive glandular atrophy, fibrosis, or secondary infection. Transoral sialolithotomy remains the preferred approach for immobile hilar or ductal giant stones when feasible, offering a gland-preserving and minimally morbid solution. Clinicians should integrate imaging characteristics, stone location, and patient symptoms—even in their absence—into individualized treatment algorithms to optimize outcomes and preserve salivary gland function.

Future research should focus on multicenter studies with larger cohorts to better characterize the clinical behavior and long-term outcomes of giant sialoliths. Additionally, incorporating compositional and biochemical analyses of the giant calculi could provide insights into their pathogenesis and support the development of preventive strategies. Establishing standardized, minimally invasive treatment protocols may also help optimize surgical decision-making and improve patient outcomes.

## 6. Conclusions

In conclusion, asymptomatic giant submandibular sialoliths are rare, more commonly observed in men, often located in Wharton’s duct, and typically managed through transoral surgical removal. Further prospective and standardized studies are needed to better elucidate their clinical characteristics, optimal diagnostic imaging approaches, and most effective treatment strategies, thereby improving patient care in both healthcare centers and private practices.

## Figures and Tables

**Table 2:** Appraisal of standardization of the selected case reports (*n* = 6). CARE Case Report Guidelines. Y: yes; N: no; Yn: absolute number of Y; Nn: absolute number of N; Y%: percentage of Y; N%: percentage of N.

Topic	Item	Checklist Item Description	Liu and Lo, 2023 [22]	Brooks et al., 2021 [1]	Abdullah and Alqudehy, 2016 [21]	Cottrell et al., 2011 [8]	Emir et al., 2010 [20]	Graziani et al., 2006 [19]
Title	1	The diagnosis or intervention of primary focus followed by the words “case report”	Y	Y	Y	Y	N	N
Key Words	2	2 to 5 keywords that identify diagnoses or interventions in this case report, including “case report”	N	N	N	N	N	N
Abstract	3a	Introduction: What is unique about this case and what does it add to the scientific literature?	N	N	N	N	N	N
	3b	Main symptoms and/or important clinical findings	Y	Y	N	Y	Y	Y
	3c	The main diagnoses, therapeutic interventions, and outcomes	N	N	Y	N	N	Y
	3d	Conclusion—What is the main “take-away” lesson(s) from this case?	Y	Y	N	N	N	Y
Introduction	4	One or two paragraphs summarizing why this case is unique (may include references)	N	N	N	N	N	N
Patient Information	5a	De-identified patient-specific information	Y	Y	Y	Y	Y	Y
	5b	Primary concerns and symptoms of the patient	Y	Y	Y	Y	Y	Y
	5c	Medical, family, and psycho-social history including relevant genetic information	N	N	N	N	N	N
	5d	Relevant past interventions with outcomes	Y	Y	Y	Y	N	N
Clinical Findings	6	Describe significant physical examination (PE) and important clinical findings	Y	Y	Y	Y	Y	Y
Timeline	7	Historical and current information from this episode of care organized as a timeline	Y	Y	Y	Y	Y	Y
Diagnostic Assessment	8a	Diagnostic testing (such as PE, laboratory testing, imaging, surveys)	Y	Y	Y	Y	Y	Y
	8b	Diagnostic challenges (such as access to testing, financial, or cultural)	N	N	N	N	N	N
	8c	Diagnosis (including other diagnoses considered)	Y	Y	Y	Y	Y	Y
	8d	Prognosis (such as staging in oncology) where applicable	N	N	N	N	N	N
Therapeutic Intervention	9a	Types of therapeutic intervention (such as pharmacologic, surgical, preventive, self-care)	Y	N	Y	Y	Y	Y
	9b	Administration of therapeutic intervention (such as dosage, strength, duration)	N	N	Y	Y	N	N
	9c	Changes in therapeutic intervention (with rationale)	N	N	N	N	N	N
Follow-up and Outcomes	10a	Clinician and patient-assessed outcomes (if available)	Y	N	Y	Y	Y	Y
	10b	Important follow-up diagnostic and other test results	Y	N	Y	Y	Y	Y
	10c	Intervention adherence and tolerability (How was this assessed?)	Y	N	Y	Y	Y	Y
	10d	Adverse and unanticipated events	N	N	N	N	N	N
Discussion	11a	A scientific discussion of the strengths AND limitations associated with this case report	N	N	N	N	N	N
	11b	Discussion of the relevant medical literature with references	Y	Y	Y	Y	Y	Y
	11c	The scientific rationale for any conclusions (including assessment of possible causes)	Y	Y	Y	Y	Y	Y
	11d	The primary “take-away” lessons of this case report (without references) in a one-paragraph conclusion	Y	Y	Y	Y	Y	Y
Patient Perspective	12	The patient should share their perspective in one to two paragraphs on the treatment(s) they received	N	N	N	N	N	N
Informed Consent	13	Did the patient give informed consent? Please provide if requested	Y	N	N	Y	N	N
		Yn %Y	18 60%	13 43.3%	17 56.7%	18 60%	14 46.7%	16 53.3%
		Nn/ %N	12 40%	17 56.7%	13 43.3%	12 40%	16 53.3%	14 46.7%

**Table 3:** Appraisal of methodological quality of the selected case reports (*n* = 6). Joanna Briggs Institute. Y: yes; N: no; -, not applicable.

Methodological Items/Selected Studies	Liu and Lo, 2023 [22]	Brooks et al., 2021 [1]	Abdullah and Alqudehy, 2016 [21]	Cottrell et al., 2011 [8]	Emir et al., 2010 [20]	Graziani et al., 2006 [19]
1. Demographic characteristics	N	N	N	N	N	Y
2. Patient’s history clearly described	Y	Y	Y	Y	Y	Y
3. Current clinical condition	Y	Y	Y	Y	Y	Y
4. Diagnostic tests or assessment methods	Y	Y	Y	Y	Y	Y
5. Intervention(s) or treatment procedure(s)	Y	-	Y	Y	Y	Y
6. Post-intervention clinical condition	Y	-	Y	Y	Y	Y
7. Adverse events (harms)	N	-	N	N	N	N
8. Take-away lessons	Y	N	N	Y	N	N
Total Questions Considered	8	5	8	8	8	8
Score	6	3	5	6	5	6
%	75	60	62.5	75	62.5	75

## Data Availability

The data presented in this study are available upon request from the corresponding author. The data are not publicly available due to the inclusion of clinical patient information.

## Supplementary Materials

The following supporting information can be downloaded at https://www.mdpi.com/article/10.3390/clinpract15110205/s1; Supplementary Material S1: Patient history, authorization for diagnosis and/or management procedures, and informed consent applied in this case report. Supplementary Material S2: Ultrasound report. Supplementary Material S3: PROSPERO registration. Supplementary Material S4: PRISMA checklist.
