# Supplementary material for: Giant Asymptomatic Submandibular Sialolith: A Case Report Accompanied by Systematic Review
Source: Clin Pract. 2025 Nov 10;15(11):205. doi: 10.3390/clinpract15110205 (PMC12651031; doi:10.3390/clinpract15110205)
Supplement: Supplementary file 1 [file clinpract-15-00205-s001.zip › Supplementary 1.pdf]

Centro Universitário do Norte de São Paulo - UNORTE

Núcleo de Ensino Odontológico - NEO

SAÚDE GERAL

Nome:

01. Está sob tratamento médico? sim ( ) não (X)

Qual(is) ? —

Medicamentos em uso: ? —

02. Teve ou tem alguma destas doenças?

Hepatite sim ( ) não (X) qual tipo? —

Diabetes sim ( ) não (X) qual tipo? —

03. Sua pressão arterial é? normal (X) baixa ( ) alta ( )

Ingere algum medicamento para a pressão? sim ( ) não (X)

Qual(is) ? —

04. Sangra muito quando se machuca? sim ( ) não (X)

05. Sua cicatrização é normal? sim (X) não ( )

06. Tem algum tipo de alergia? sim ( ) não (X)

Alergia a que? —

07. Apresentou algum problema com anestésico? sim ( ) não (X)

Qual? —

08-Apresentou alguma reação adversa com medicamento? sim ( ) não (X)

Qual? —

09. Faz uso de drogas? sim ( ) não (X)

10. É fumante? sim ( ) não (X)

11. Está grávida? sim ( ) não (X)

12. Algum problema cardíaco? sim ( ) não (X)

Qual? —

13. Já fez tratamento para alguma enfermidade grave? sim ( ) não (X)

Qual? —

14. Já foi internado para tratamento médico? sim ( ) não (X)

Qual? —

15. Alguma observação sobre sua saúde geral que gostaria de informar?

N.D.N

Assinatura: Data: 06/06/2025

Centro Universitário do Norte de São Paulo - UNORTE

Núcleo de Ensino Odontológico - NEO

AUTORIZAÇÃO PARA DIAGNÓSTICO E/OU EXECUÇÃO DE TRATAMENTO

PACIENTE: —

DATA DE NASCIMENTO: — / — / —

RESPONSÁVEL: —

ENDEREÇO: —

FONE: —

ALUNO: Lucas

ESPECIALIDADE: Cirurgia

Por este instrumento de autorização por mim assinado, dou pleno consentimento ao Centro Universitário do Norte de São Paulo - UNORTE para, por intermédio de seus professores, assistentes e alunos devidamente autorizados, fazer quaisquer diagnóstico, planejamento e tratamento em minha pessoa ou meu filho menor de idade, de acordo com os conhecimentos enquadrados no campo dessa especialidade.

Concordo também, que todas as radiografias, fotografias, modelos, desenhos, históricos de antecedentes familiares, resultados de exames clínicos e de laboratório bem como, quaisquer outras informações concernentes ao planejamento de diagnóstico e/ou tratamento, constituem propriedade exclusiva desta escola, à qual dou pleno direitos de retenção, uso para quaisquer fins de ensino e de divulgação em jornais e/ou revistas científicas do País e do exterior.

São José do Rio Preto, 06 de junho de 2025.

....

Assinatura do paciente ou responsável

....

R.G.

Centro Universitário do Norte de São Paulo - UNORTE
Núcleo de Ensino Odontológico - NEO

CONSENTIMENTO PARA CIRURGIA ORAL E ANESTESIA

Este é meu consentimento para ....
E qualquer participante do curso de especialização, a proceder ao seguinte tratamento ou cirurgia ou o que for proposto:

Como previamente explicado para mim ou outros procedimentos que se refizer necessário ou indicado no meu plano operatório. Entendo que o propósito do procedimento é tratar e corrigir minhas deformidades e doenças buco-maxilo-faciais, que podem ocorrer edema, inchaço, dor, infecção, formação de abscessos, doenças periodontais, má-oclusão, fraturas da mandíbula e maxila, perda de dentes, perda óssea, sangramento, traumatismo aos dentes vizinhos, perda de restaurações, rachaduras dos lábios, restrição da abertura bucal por dias ou semanas, traumatismo do nervo mandibular, o que pode resultar em dormência ou pontadas nos lábios, queixo, gengiva, dentes e língua no lado operado, que pode persistir por dias, semanas, meses, e em condições remotas, permanentemente.

Comunicação buco-sinusal que pode requerer cirurgias corretivas adicionais.

Aceito seguir as recomendações pós-operatórias, como alimentação, prescrição de remédios e cuidados locais e gerais, permito administração de anestesia local, sedação ou anestesia geral se for necessário. Estou ciente que estas drogas podem produzir tonturas, falta de coordenação e confusão mental, o uso de álcool ou outras drogas podem aumentar estes efeitos.

Estou sendo aconselhado a não dirigir veículos ou realizar trabalhos perigosos enquanto estiver sob os efeitos destas drogas, que podem perdurar até 24 horas após a administração ou ingestão dos mesmos.

Entendo que estes anestésicos podem provocar sérios envolvimentos orgânicos e que são inerentes em qualquer procedimento que requer anestesia local sedação ou anestesia geral.

Se qualquer condição suceder no curso da operação ou do meu tratamento, que dependa da decisão do professor, doutor ou assistentes do curso os quais esteja especificado aqui nestes termos, eu solicito e dou total e ampla autorização para que se decida qualquer procedimento que se aplique e se julgue necessário.

Tive a aportunidade de expor e discutir meu passado medica, a editoria da minha saúde, incluindo riscos ou problemas sérios.

O sucesso do meu tratamento depende da cumplicidade das instruções do operador. Eu aceito cooperar completamente com as recomendações dos doutores e assistentes, enquanto estiver sob seus cuidados.

Eu certifico que li e compreendi os termos e palavras deste termo de consentimento.

....	....
Paciente, pai ou responsável	Testemunha
Lucas<br>..	....
Doutor	Testemunha
