# Supplementary material for: Giant Asymptomatic Submandibular Sialolith: A Case Report Accompanied by Systematic Review
Source: Clin Pract. 2025 Nov 10;15(11):205. doi: 10.3390/clinpract15110205 (PMC12651031; doi:10.3390/clinpract15110205)
Supplement: Supplementary file 1 [file clinpract-15-00205-s001.zip › Supplementary 2.pdf]

partmed
saúde e medicina

Nome:

Data: 22/05/2025

ULTRASSONOGRAFIA DE CERVICAL

Técnica do exame:
Exame realizado com transdutor linear multifrequencial:

Relatório:
Glândula submandibular direita de contornos normais, dimensões discretamente aumentadas em relação à contralateral, e ecotextura finamente heterogênea. Nota-se ainda imagem ovalar, hiperecogênica, determinante de sombra acústica posterior localizada na topografia distal do ducto submandibular direito (Wharton), medindo 2,0 x 0,6 x 0,6 cm nos seus maiores eixos, com dilatação ductal correspondente.
Glândulas parótidas e submandibular esquerda com contornos regulares, ecotextura sólida e homogênea com dimensões normais.
Glândula tireoide de aspecto habitual.
Observam-se linfonodos de aspecto habitual nas cadeias cervicais.

IMPRESSÃO:

- Sinais ecográficos sugestivos de sialolitíase à direita.
- Demais estruturas cervicais avaliadas de aspecto ecográfico habitual.

Alfino José Costa Jr.

Dr. Alfino José Costa Junior

CRM/SP: 228.947

Rua Benjamim Constant, Nº 3435, Vila Imperial – Próximo ao Colégio London
Cep: 15015-600 São José do Rio Preto
