# Supplementary material for: Giant Asymptomatic Submandibular Sialolith: A Case Report Accompanied by Systematic Review
Source: Clin Pract. 2025 Nov 10;15(11):205. doi: 10.3390/clinpract15110205 (PMC12651031; doi:10.3390/clinpract15110205)
Supplement: Supplementary file 1 [file clinpract-15-00205-s001.zip › Supplementary 3.pdf]

NIHR | National Institute for Health and Care Research

PROSPERO
International prospective register of systematic reviews

Giant Asymptomatic Submandibular Sialolith: A case report and systematic review

Gestter Lattari Tessarin, Renato Gomes Azevedo, Julia da Conceição Francisquini

Citation
Gestter Lattari Tessarin, Renato Gomes Azevedo, Julia da Conceição Francisquini. Giant Asymptomatic Submandibular Sialolith: A case report and systematic review. PROSPERO 2025 CRD420251076737. Available from https://www.crd.york.ac.uk/PROSPERO/view/CRD420251076737.

REVIEW TITLE AND BASIC DETAILS

Review title
Giant Asymptomatic Submandibular Sialolith: A case report and systematic review

Condition or domain being studied
Asymptomatic; Submandibular Sialolithiasis; Examination Of Submandibular Gland; Removal Of Calculus From Submandibular Duct; Salivary Calculus; Sialolithiasis; Sialoadenitis Of The Submandibular Gland; Submandibular Calculus; Manipulative Removal Of Calculus From Submandibular Duct; Open Extraction Of Calculus From Submandibular Duct; Open Operations On Submandibular Duct; Injury Of Salivary Duct

Rationale for the review
The review is being carried out, in principle, because there is no systematic review of the literature that describes the clinical conditions, sex and age prevalence, clinical procedures adopted for resolution, and outcome of large/unusual asymptomatic sialoliths of the submandibular gland.

Review objectives
This review aims to answer the following questions:

What is the best clinical approach for asymptomatic submandibular sialoliths?

What is the incidence of this pathology?

What is the prevalence of this pathology?

What are the outcomes of this pathology?

What is the mineral/histological composition of this pathology?

What are the main drugs administered in this pathology?

What are the outcomes of this pathology?

What are the main surgical techniques adopted for this pathology?

What is the best clinical approach for asymptomatic submandibular sialoliths?

Keywords
Asymptomatic; GIANT SIALOLITH; Submandibular gland; Wharton's duct; Case report

Country
Brazil

ELIGIBILITY CRITERIA

Population

Included
Patients (adults) with asymptomatic giant sialoliths located in the submandibular gland and/or Wharton's duct.

Excluded
Patients with simptomatic sialolith located in the submandibular gland and/or Wharton's duct; patients with sialolith into the sublingual or parotid glands; patients under 18 years old.

Intervention(s) or exposure(s)

Included
Excision Of Salivary Gland; Removal Of Salivary Stone; Incision Of Salivary Duct; Examination Of Submandibular Gland; Open Extraction Of Calculus From Submandibular Duct; Drug Therapy

Comparator(s) or control(s)
This review does not have any comparators

Study design
Only nonrandomized study types will be included.

Included
Case reports.

Excluded
Manuscripts not in English; conference abstracts; case reports that observed painful symptoms; sialoliths smaller than 15 mm.

Context
This systematic review will include case reports only. This selection was carried out to discuss the main drug administrations, images for diagnosis, surgical intervention, and others (check the objective to review).

TIMELINE OF THE REVIEW

Date of first submission to PROSPERO
04 July 2025

Review timeline

Start date: 4 July 2025. End date: 30 August 2025.

Date of registration in PROSPERO

04 July 2025

AVAILABILITY OF FULL PROTOCOL

Availability of full protocol

A full protocol has been written and uploaded to PROSPERO. The protocol may be accessed through this link https://www.crd.york.ac.uk/PROSPEROFILES/bb3037d02b11bc20572309c295bdc414.pdf.

SEARCHING AND SCREENING

Search for unpublished studies

Only published studies will be sought.

Main bibliographic databases that will be searched

The main databases to be searched are *CENTRAL - Cochrane Central Register of Controlled Trials*, *Embase - Embase via Ovid*, *Embase.com*, *MEDLINE* and *PubMed*.

Search language restrictions

The review will only include studies published in English.

Search date restrictions

There are no search date restrictions.

Other methods of identifying studies

No other methods will be used.

Link to search strategy

A full search strategy has been uploaded to PROSPERO. The PDF may be accessed through this link https://www.crd.york.ac.uk/PROSPEROFILES/7c87622f94a113e9b5cf0a86000ae912.pdf.

Selection process

Studies will be screened independently by at least two people (or person/machine combination) with a process to resolve differences.

Other relevant information about searching and screening

None

DATA COLLECTION PROCESS

Data extraction from published articles and reports

Data will be extracted independently by at least two people (or person/machine combination) with a process to resolve differences.

Authors will not be contacted for further information.

Study risk of bias or quality assessment

Risk of bias will be assessed using:

Quality of studies: CAse REport (CARE) Guidelines Checklist

Risk of Bias: Joanna Briggs Institute (JBI) Critical Appraisal Checklists for Case Reports and Case Series

Data will be assessed independently by at least two people (or person/machine combination) with a process to resolve differences.

Additional information will be sought from study investigators if required information is unclear or unavailable in the study publications/reports.

Reporting bias assessment

Risk of bias due to missing results will not be assessed

Certainty assessment

CAse REport (CARE) Guidelines Checklist

OUTCOMES TO BE ANALYSED

Main outcomes

Chirurgical technique adopted

Sex and age prevalence

Side involved

Images for diagnosis

management.

Additional outcomes

There are no additional outcomes.

PLANNED DATA SYNTHESIS

Strategy for data synthesis

The formal synthesis following the steps below:

Qualitative systematic review

Data will be presented in a number of different ways. A typical procedure in the health sciences is thematic analysis.

As explained by James Thomas and Angela Harden (2008) in an article for BMC Medical Research Methodology:

"Thematic synthesis has three stages:

a. the coding of text 'line-by-line'

b. the development of 'descriptive themes'

c. generation of 'analytical themes'

CURRENT REVIEW STAGE

Stage of the review at this submission

Review stage	Started	Completed
Pilot work		
Formal searching/study identification		
Screening search results against inclusion criteria		
Data extraction or receipt of IPD		
Risk of bias/quality assessment		
Data synthesis		

Review status
The review is currently planned or ongoing.

Publication of review results
Results of the review will be published in English.

REVIEW AFFILIATION, FUNDING AND PEER REVIEW

Review team members
Professor Gestter Lattari Tessarin (review guarantor and contact) Unesp. Brazil.

No conflict of interest declared.

Professor Renato Gomes Azevedo. Northern Sao Paulo University Center. Brazil.

No conflict of interest declared.

Miss Julia da Conceição Francisquini. Northern Sao Paulo University Center. Brazil.

No conflict of interest declared.

Named contact
Professor Gestter Lattari Tessarin (gestter.tessarin@unesp.br). Unesp. Brazil.

Review affiliation
São Paulo State University and Northern São Paulo University Center

Funding source
Review has no funding and no agreed support from an academic institution and is done in authors’ own time.

Peer review

My review protocol was revised by blind examination and an expert professor.

Funding application: not applied.

ADDITIONAL INFORMATION

Review conflict of interest

Declared individual interests are recorded under team member details.. No additional interests are recorded for this review.

Medical Subject Headings

Humans; Incidence; Minerals; Prevalence; Salivary Duct Calculi; Salivary Gland Calculi

SIMILAR REVIEWS

Check for similar records already in PROSPERO

After checking the list of possible similar reviews in PROSPERO and also carrying out an extensive search on scientific research websites, no systematic review as proposed was found.

PROSPERO version history

- Version 1.0, published 04 Jul 2025

Disclaimer

The content of this record displays the information provided by the review team. PROSPERO does not peer review registration records or endorse their content.

PROSPERO accepts and posts the information provided in good faith; responsibility for record content rests with the review team. The guarantor for this record has affirmed that the information provided is truthful and that they understand that deliberate provision of inaccurate information may be construed as scientific misconduct.

PROSPERO does not accept any liability for the content provided in this record or for its use. Readers use the information provided in this record at their own risk.

Any enquiries about the record should be referred to the named review contact
